# Supplementary material for: Superconducting diode effect via conformal-mapped nanoholes
Source: Nat Commun. 2021 May 11;12:2703. doi: 10.1038/s41467-021-23077-0 (PMC8113273; doi:10.1038/s41467-021-23077-0)
Supplement: Supplementary file 1 — Supplementary Information [file 41467_2021_23077_MOESM1_ESM.pdf]

Supplementary Information

Superconducting diode effect via conformal-mapped nanoholes

Yang-Yang Lyu[1,2,+], Ji Jiang[3,4,+], Yong-Lei Wang[1,*], Zhi-Li Xiao[2,5,*], Sining Dong[1], Qing-Hu Chen[3], Milorad V. Milošević[4], Huabing Wang[1,6], Ralu Divan[7], John E. Pearson[2], Peiheng Wu[1,6], Francois M. Peeters[4] and Wai-Kwong Kwok[2]

[1]*Research Institute of Superconductor Electronics, School of Electronic Science and Engineering, Nanjing University, Nanjing 210023, China*

[2]*Materials Science Division, Argonne National Laboratory, Argonne, Illinois 60439, USA*

[3]*Department of Physics, Zhejiang University, Hangzhou, China*

[4]*NANOlab Center of Excellence and Department of Physics, University of Antwerp, Antwerp, Belgium*

[5]*Department of Physics, Northern Illinois University, DeKalb, Illinois 60115, USA*

[6]*Purple Mountain Laboratories, Nanjing 211111, China*

[7]*Center for Nanoscale Materials, Argonne National Laboratory, Argonne, Illinois 60439, USA*

+ Authors contribute equally

* Correspondence to: yongleiwang@nju.edu.cn; xiao@anl.gov;

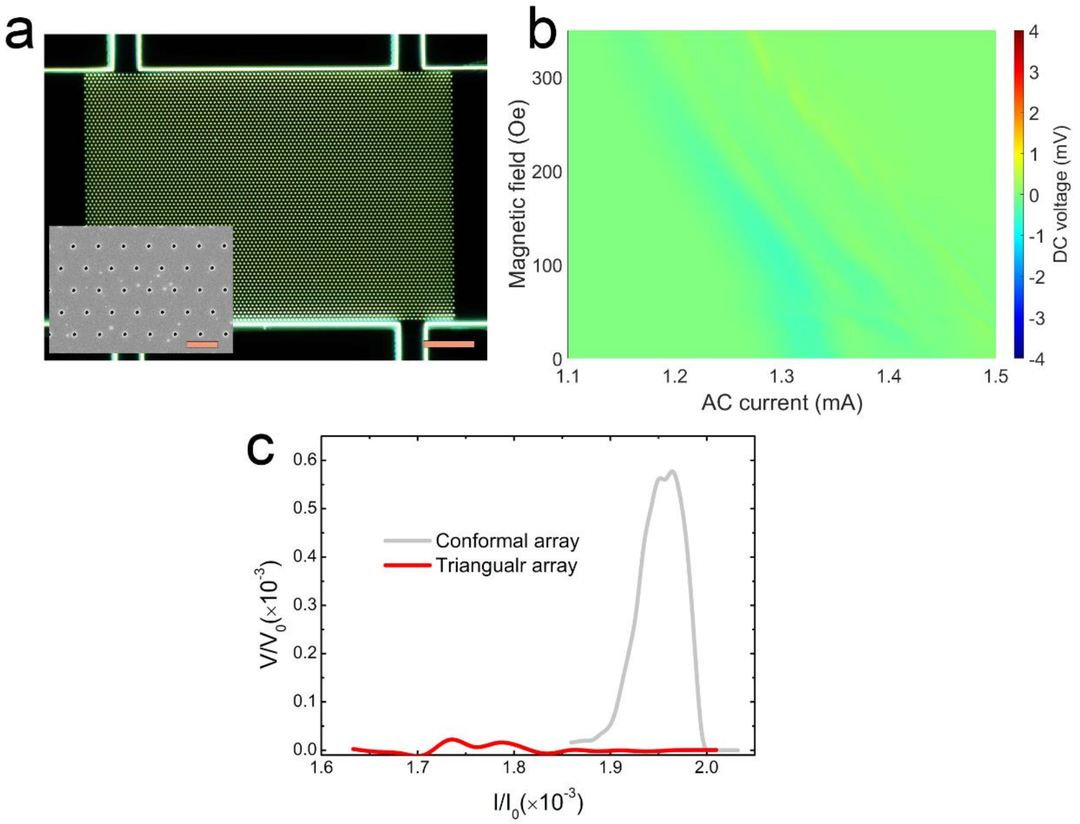

Supplementary Fig. 1 | Results for section TRI with triangular array of nanoholes. **a**, Dark-field optical image of the TRI section in Fig. 1a. Scale bar, 10 μm. The inset is a SEM image of the nanoholes with a nominal diameter of 110 nm. Scale bar of inset, 1 μm. **b,** Color map (experimental results) of the magnetic field and AC current dependent DC voltages obtained from section TRI, displaying negligible rectification. **c,** Simulated rectification signals for a sample with nano-holes in a triangular array. The rectification is nearly absent compared to the sample with nanoholes in a conformal array, and consistent with the experimental observation.

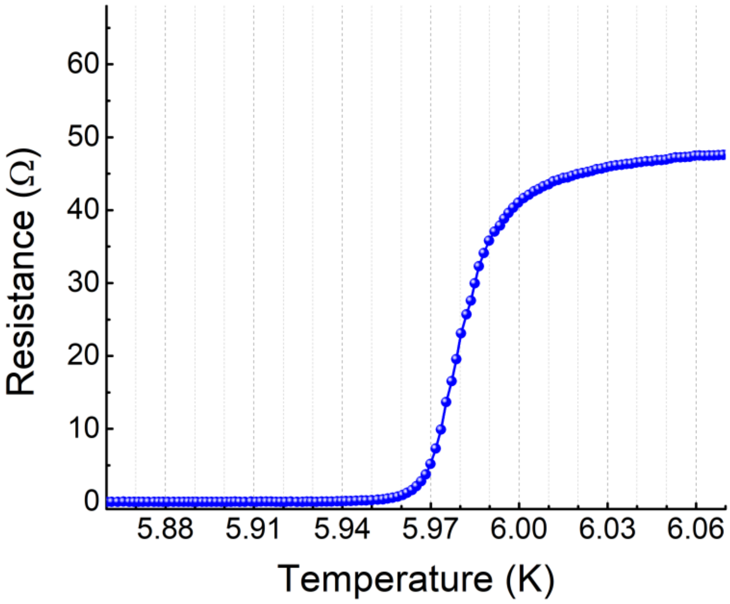

Supplementary Fig. 2 | Superconducting transition of the MoGe film. The resistance versus temperature curve shows an onset transition temperature of ~6 K.

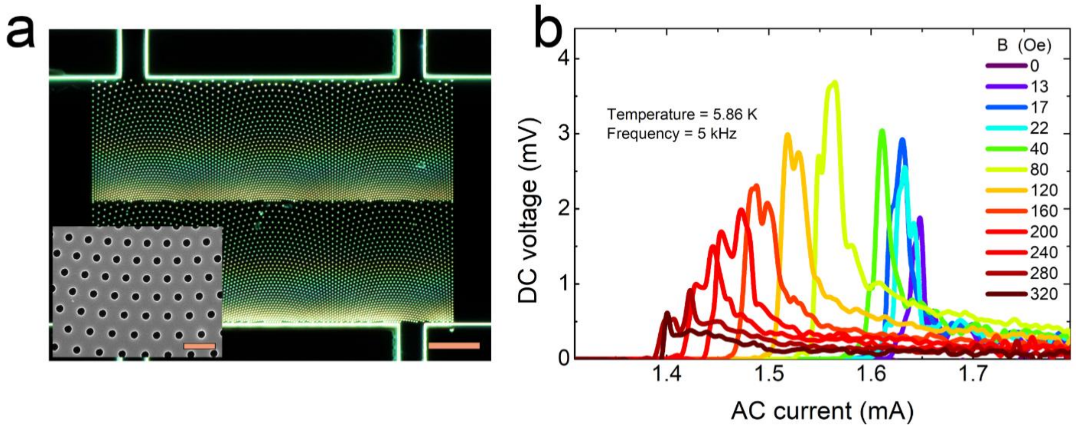

Supplementary Fig. 3 | Rectification effect for a sample with a nominal hole diameter of 220 nm. a, Dark field optical image of the sample. Scale bar, 10 μm. The inset is a SEM image of the nanoholes with diameter of ~220 nm. Scale bar of inset, 1 μm. **b,** Curves of the magnetic field and AC current dependent DC voltages. The maximal rectification signal occurs around the magnetic field of ~80 Oe, which corresponds to a flux-quantum density that is twice the average hole density.

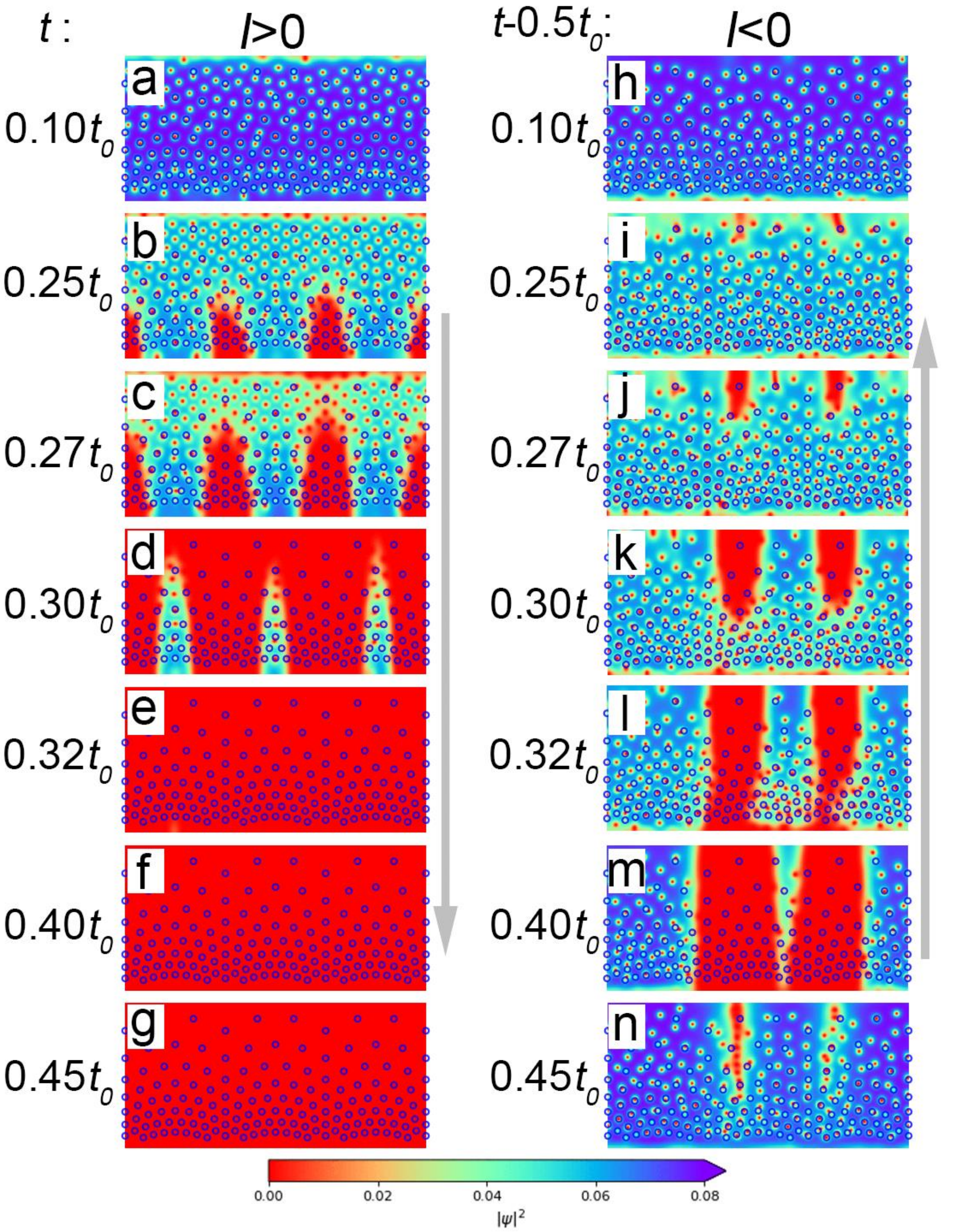

Supplementary Fig. 4 | Nonreciprocal superconducting-to-normal transitions. Screenshots of superconducting Cooper-pair density from Video 1 at various instances t (t_0 is the period of the sine wave AC current). **a-g**, in 1st half period with I>0. **h-n**, in the 2nd half period with I<0. Blue indicates superconducting state, while red indicates normal state. Gray arrows denote the direction of flux motion.

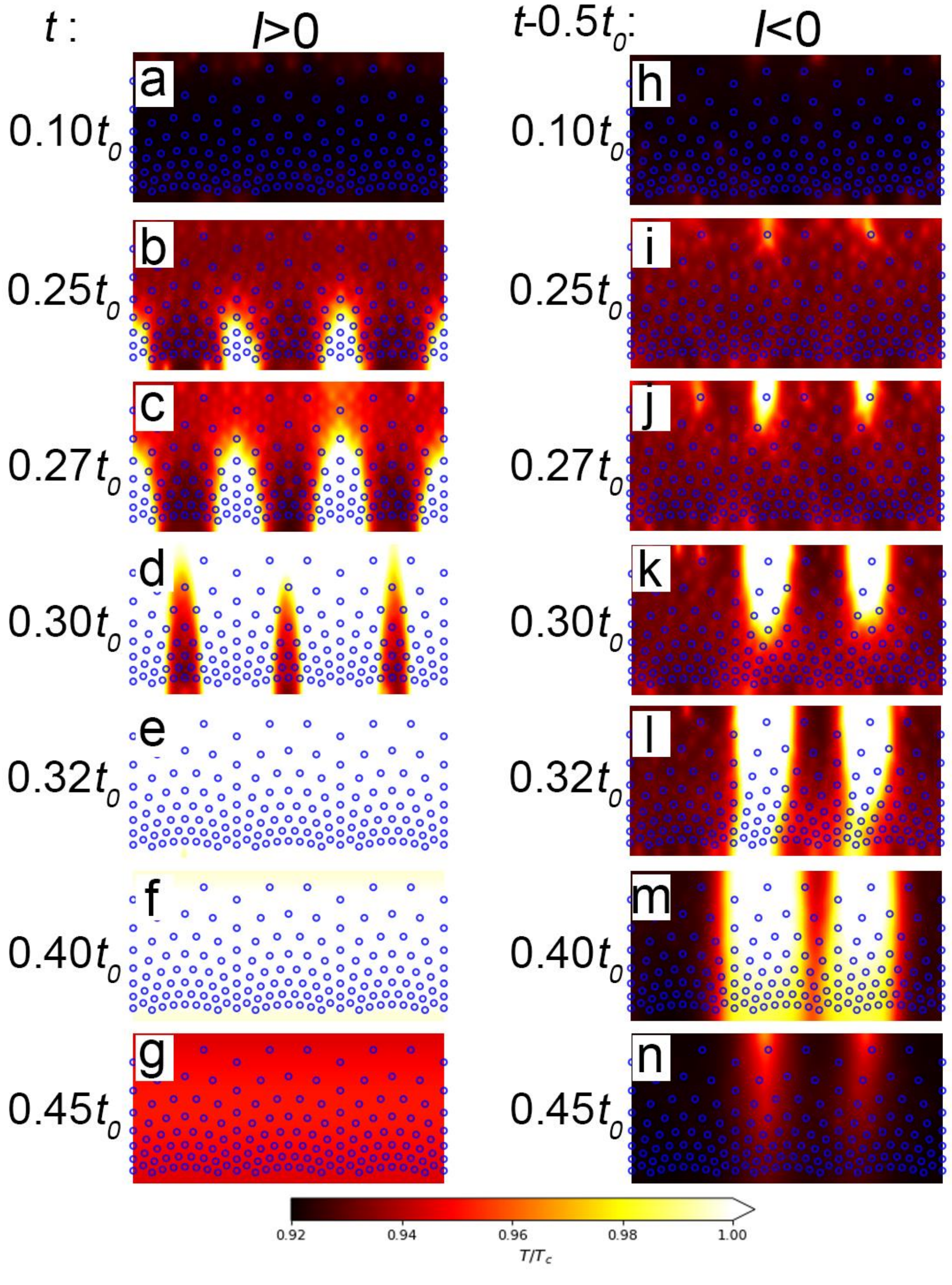

Supplementary Fig. 5 | Nonequivalent nucleation and evolution of hot spots. Screenshots of temperature maps obtained from Video 2 at various instances t (t_0 is the period of sine wave AC current). **a-g**, in the 1st half period with I>0. **h-n**, in the 2nd half period with I<0. White indicates high temperature, while dark color indicates low temperature.

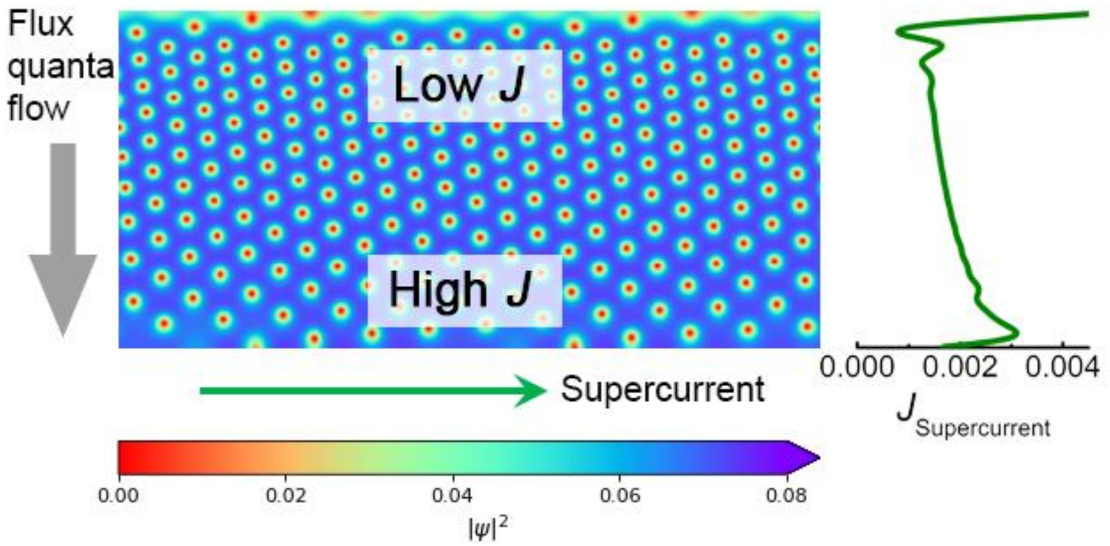

Supplementary Fig. 6 | Dynamic distribution of flux-quanta and asymmetric supercurrent density in a pristine sample. The dynamic distribution mapping of flowing flux-quanta driven by supercurrent is obtained from a screenshot of Video 3. Green and gray arrows indicate the direction of current and flux-flow, respectively. The bottom region of the fluxonic map has a higher supercurrent density, a faster flux-flow with a lower flux density as compared to those in the top region. Right panel depicts distribution of supercurrent density along the vertical direction.

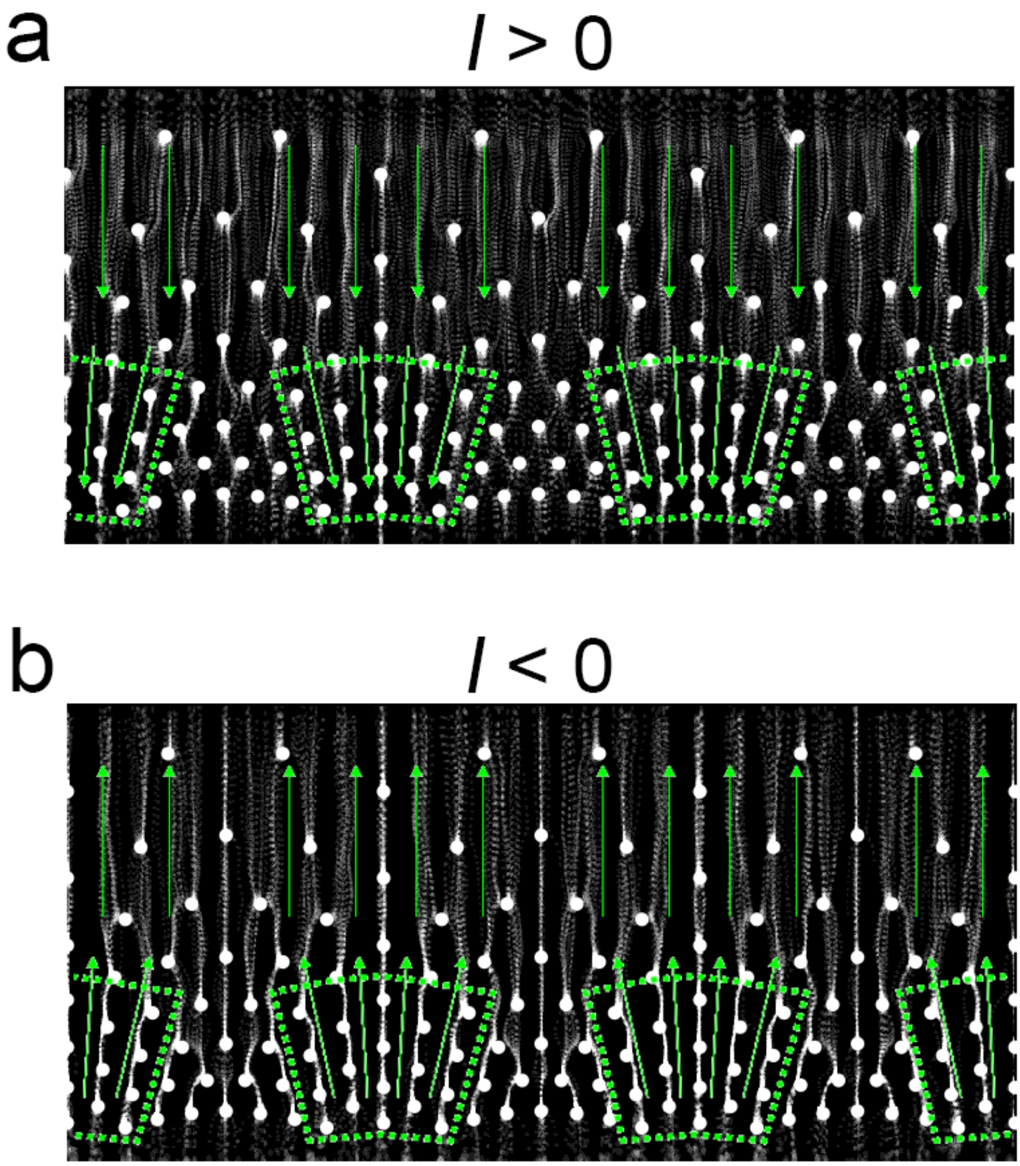

Supplementary Fig. 7 | Trajectories of flux-quanta. a and **b,** fluxonic trajectory map under positive (a) and negative (b) current, respectively. White dots show the positions of the nanoholes. White lines are single flux-quantum trajectories. Green arrows depict the direction of flux-quantum motion. The dotted fans highlight the areas with strong flux funneling effect.

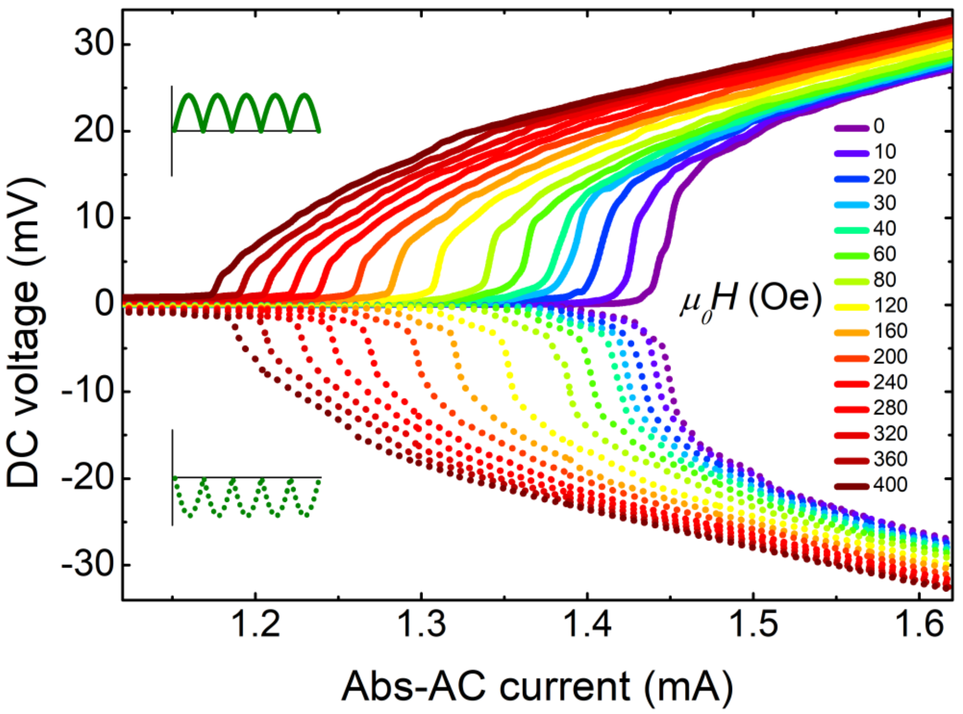

Supplementary Fig. 8 | Magnetic field dependent quasi-DC experiments. The measurements were conducted using quasi-DC currents with Abs-AC waveforms shown in the insets.

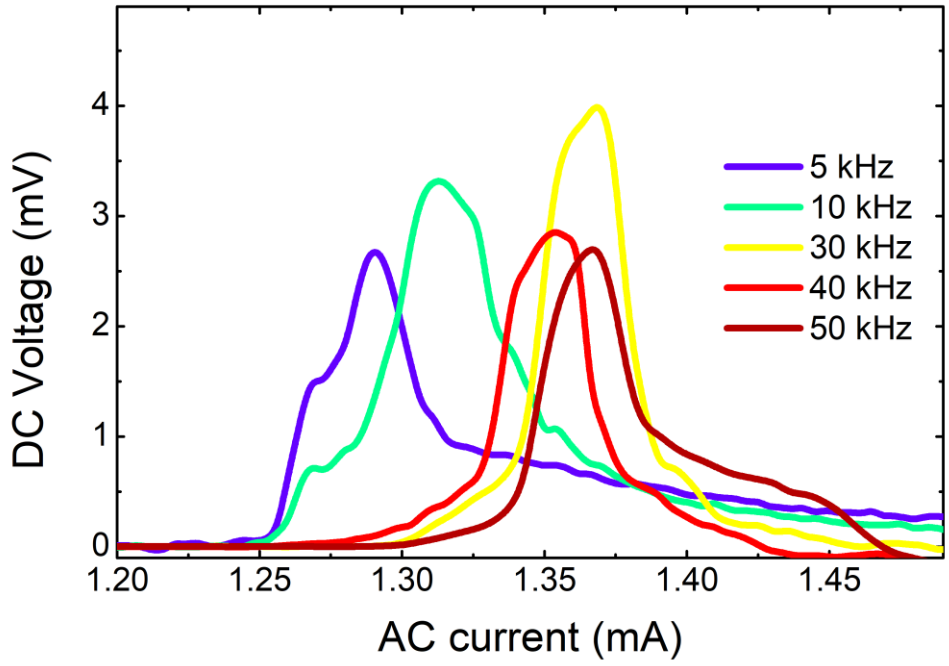

Supplementary Fig. 9 | Frequency dependence of the superconducting diode effect.

Rectification signals obtained at various AC frequencies. The magnetic field is 40 Oe.
