# Supplementary material for: Superconducting diode effect via conformal-mapped nanoholes
Source: Nat Commun. 2021 May 11;12:2703. doi: 10.1038/s41467-021-23077-0 (PMC8113273; doi:10.1038/s41467-021-23077-0)
Supplement: Supplementary file 3 — Description of Additional Supplementary Files [file 41467_2021_23077_MOESM3_ESM.pdf]

Description of Additional Supplementary Files

Supplementary Movie 1 | Nonequivalent evolution of the superconducting condensate in a single period of sine-wave AC current. Black dots are nanoscale holes in a conformal array. Blue indicates superconducting state, while red indicates normal state. Gray arrows denote directions of flux-quantum motion. In the 1st half-cycle with $I>0$, the sample changes from the superconducting state into a complete normal state (with zero order parameter), while in the 2nd half-cycle with $I<0$, the sample cannot fully reach the normal state.

Supplementary Movie 2 | Nonequivalent temperature evolution in a single period of sine-wave AC current. Video showing nonequivalent nucleation and evolution of hot spots with positive and negative current. Blue circles are nanoscale holes in a conformal array.

Supplementary Movie 3 | Dynamic motion of flux-quanta driven by current in a pristine sample exposed to magnetic field. Red dots (with zero order parameter) show flux-quanta. Video showing a dynamic gradient distribution of flux-quanta.
