# Supplementary figures and images for: Superconducting diode effect via conformal-mapped nanoholes
Source: Nat Commun. 2021 May 11;12:2703. doi: 10.1038/s41467-021-23077-0 (PMC8113273; doi:10.1038/s41467-021-23077-0)

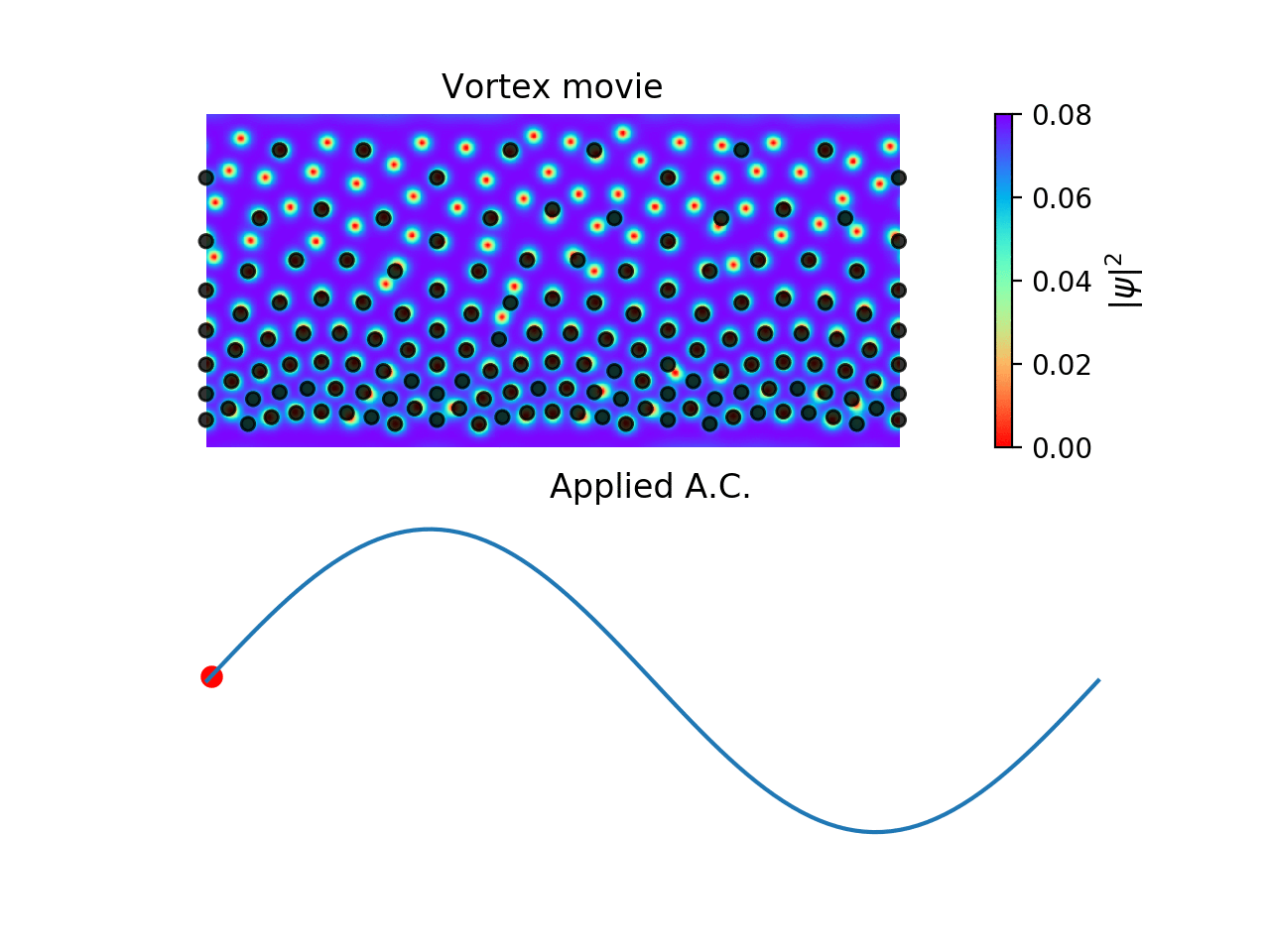

Supplement: Supplementary file 4 — Supplementary Movie 1 [file 41467_2021_23077_MOESM4_ESM.gif]

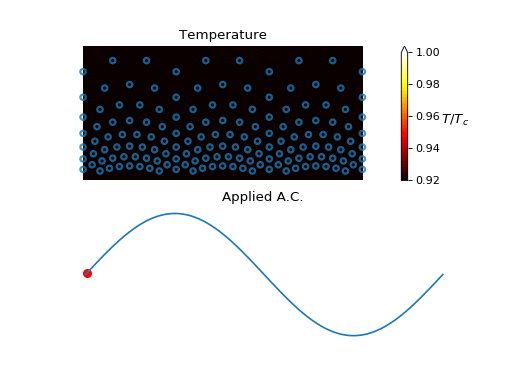

Supplement: Supplementary file 5 — Supplementary Movie 2 [file 41467_2021_23077_MOESM5_ESM.gif]

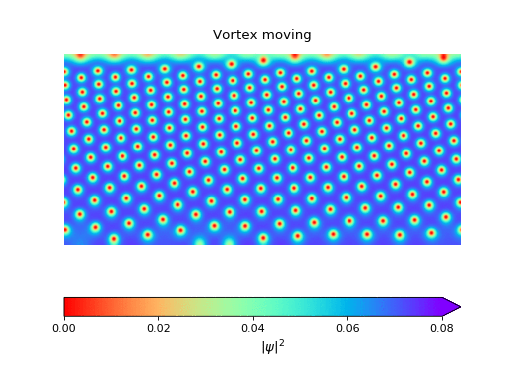

Supplement: Supplementary file 6 — Supplementary Movie 3 [file 41467_2021_23077_MOESM6_ESM.gif]
